# Understanding ‘monitoring’ data–the association between measured stressors and athlete responses within a holistic basketball performance framework

**DOI:** 10.1371/journal.pone.0270409

**Published:** 2022-06-24

**Authors:** Richard A. J. Mercer, Jennifer L. Russell, Lauren C. McGuigan, Aaron J. Coutts, Donnie S. Strack, Blake D. McLean

**Affiliations:** 1 Faculty of Health, School of Sport, Exercise and Rehabilitation, University of Technology Sydney (UTS), Sydney, NSW, Australia; 2 Human and Player Performance, Oklahoma City Thunder Professional Basketball Club, Oklahoma City, Oklahoma, United States of America; Universita degli Studi di Milano, ITALY

## Abstract

This study examined associations between cumulative training load, travel demands and recovery days with athlete-reported outcome measures (AROMs) and countermovement jump (CMJ) performance in professional basketball. Retrospective analysis was performed on data collected from 23 players (mean±SD: age = 24.7±2.5 years, height = 198.3±7.6 cm, body mass = 98.1±9.0 kg, wingspan = 206.8±8.4 cm) from 2018–2020 in the National Basketball Association G-League. Linear mixed models were used to describe variation in AROMs and CMJ data in relation to cumulative training load (previous 3- and 10-days), hours travelled (previous 3- and 10-day), days away from the team’s home city, recovery days (i.e., no travel/minimal on-court activity) and individual factors (e.g., age, fatigue, soreness). Cumulative 3-day training load had negative associations with fatigue, soreness, and sleep, while increased recovery days were associated with improved soreness scores. Increases in hours travelled and days spent away from home over 10 days were associated with increased sleep quality and duration. Cumulative training load over 3 and 10 days, hours travelled and days away from home city were all associated with changes in CMJ performance during the eccentric phase. The interaction of on-court and travel related stressors combined with individual factors is complex, meaning that multiple athletes response measures are needed to understand fatigue and recovery cycles. Our findings support the utility of the response measures presented (i.e., CMJ and AROMs), but this is not an exhaustive battery and practitioners should consider what measures may best inform training periodization within the context of their environment/sport.

## Introduction

When athletes are to perform at their best, practitioners working with these athletes must consider a range of stressors encountered during the season, and how individuals respond to those demands. During a professional basketball season, players experience physical loading frequently during games and practices, combined with extensive travel across multiple time zones and limited recovery days (i.e., days without travel and minimal to no on-court activity) [1]. However, there is a paucity of information describing the association between physical demands in professional basketball and an athlete’s physical readiness to perform during competition.

In basketball, there are requisite physical capacities that are necessary to sustain the technical and tactical aspects of the sport, which ultimately determine individual and team performance outcomes. Given the congested competition and travel schedules in professional basketball, enhancing physical readiness necessitates carefully planned and well executed training, recovery, and travel [2, 3]. In this context, recovery represents processes that result in an athlete’s renewed ability to meet or exceed previous performance levels following training and competition [4]. Adequate rest from strenuous exercise is critical component in this recovery cycle, but opportunities for dedicated ‘recovery days’ are limited throughout the professional basketball season. Therefore, it may be difficult for players to fully recover from the accumulated physical and psychological stress related to games [5]. A further challenge for practitioners working in team sports is managing training prescription for many individual athletes, who possess a range of different individual characteristics (e.g., physical qualities, psychological and emotional states, age, training age, injury history, experience), with varied contextual factors (e.g., travel direction, travel duration, activity, training type, competition schedule). All of these components may each individually affect how players respond to on- and off-court stressors [6], and responses can also be measured via many different assessments (e.g., neuromuscular status, perceived wellness, heart rate variability, biomechanical measures or cognitive tests [7]). Given these challenges, practitioners commonly collect a combination of objective (e.g., countermovement jump (CMJ) tests [8]) and subjective (e.g. athlete-reported outcome measures (AROMs) [9]) assessments in an effort to measure an athlete’s physical readiness and to prescribe individual periodization strategies [6]. Indeed, the systematic collection and evaluation of ecologically valid data can be used to inform future decision-making, which is a critical element of continually improving the effectiveness of the training process [10]. In basketball, information regarding associations between training load [i.e., the training stimulus induced by both training sessions and competition [11]), travel, recovery days and training effect measures (e.g., functional, subjective, physiological, biomechanical and other measures [12]) is limited. Enhancing understanding of these relationships would facilitate better understanding and implementation of athlete monitoring processes and allow practitioners to make well-informed decisions that lead to a higher level of athlete care [1].

Whilst athlete response measures aim to quantify the response to a range of factors, many of which are relatively uncontrollable in team sport settings (e.g., playing and travel schedule, social influences), practitioners also control many elements which affect athlete responses. Indeed, it has been suggested that more emphasis should be placed on the design and implementation of sensitive and responsive training systems, in order to optimize individualization and develop context-specific training-planning solutions [10]. This requires practitioners to respond to emerging information [10], such as athlete response measures assessed regularly throughout the season [12]. Adding further complexity, training effects may respond over acute or chronic time periods and have a positive or negative impact, and subsequent performance is the resultant balance between the positive and/or negative adaptation [12]. Similarly, there is a reciprocal interaction between individual and contextual factors and training effects. For example, negative training effects (e.g., increased fatigue or poor sleep) can act as individual factors that influence the internal training load for the athlete in the following training sessions (a further description of this concept can be found in the work by Jeffries et al. [12]). To better inform the interplay of multiple inputs and outcome measures, conceptual frameworks have recently been proposed as a tool to inform context specific training monitoring [12]. This process involves identifying measurable components and their role within the training process, thereby allowing practitioners to better understand what to measure and why these components may be important [12]. This approach leads to an improved understanding that reflects the challenging nature of the environment, and these conceptual frameworks can act as a reference operational guide in practical settings [12], against which experience, observations, data and decisions can be contextualized [10].

Therefore, the aim of this work is to determine associations between short- (i.e., 3-day) and medium-term (i.e., 10-day) cumulative training load, short- and medium-term travel demands, recovery days, and individual factors (e.g., fatigue, soreness, age) with AROMs and CMJ measures over the course of a professional basketball season. Subsequently, we aim to develop a conceptual framework that describes the important inputs and outcomes in professional basketball and informs the interpretation of physical training measures within this environment.

## Methods

### Study design

For the first part of this study, a retrospective, descriptive, observational design was followed using data recorded from one team over the course of the 2018–2019 and 2019–2020 National Basketball Association (NBA) G-League seasons (October to March). The team selected was a convenience sample, as members of the research team are full-time staff members with the partner organization. The regular monitoring process of the team included collection of AROMs on home practice days, CMJ data, training load data for all on-court activity (e.g., practice and games) and travel demands for all trips away from the team’s home city throughout the season. Linear mixed models were used to determine associations between these physical demands, individual factors (e.g., age, fatigue, soreness) and training effect measures collected throughout the season.

We then developed a conceptual framework to facilitate the validation and interpretation of these physical training measures [12]. This included theorizing components of external load (i.e., physical demands associated with the training process and competition schedule) and internal load (i.e., psycho-physiological stress experienced by the athlete during the training process and competition schedule), combined with individual and contextual factors that can influence subsequent training effects in professional basketball [12]. We then identified training effect measures that are commonly used in professional basketball settings, and performance outcomes specific to basketball competition.

### Participants

Players were only included in the analysis if they met all the following criteria: i) were on the team’s roster for at least 4 weeks ii) completed 3 or more CMJ assessments and iii) completed 10 or more AROM questionnaires. Final analysis included 23 professional basketball players (mean ± SD: age = 24.7 ± 2.5 years [range: 20–30 years], height = 198.3 ± 7.6 cm, body mass = 98.1 ± 9.0 kg, wingspan = 206.8 ± 8.4 cm). This research was approved by the University of Technology Sydney (UTS) Human Research Ethics Committee (ETH19-3359), and consent was granted by the NBA and NBA Players Association as per the guidelines and requirements for ‘NBA related health research’ governed by the NBA Collective Bargaining Agreement [13].

### Procedures

#### External load measures

Training load data (i.e., PlayerLoad™) was collected from all on-court activities (e.g., individual workouts, practice, shootarounds and games) with an inertial measurement unit (IMU), sampling at 100 Hz (Catapult T6, Catapult Sports, Melbourne Australia), placed between the scapulae of the players in a customized pouch on a fitted vest. Each player wore the same IMU throughout the season to minimize inter-unit error. The validity and reliability of measuring team sport 3-dimensional movements via triaxial accelerometer has been shown previously for indoor environments [14, 15]. After all on-court activities, the units were downloaded using proprietary software (Openfield 1.12.2, Catapult Sports, Melbourne Australia) and individual training load data for each player was exported and placed in a customized Microsoft Excel spreadsheet for analysis (Microsoft, Redmond, WA, USA. For individual sessions during the season where players did not wear an IMU, training load data were estimated by multiplying the measured ‘active’ duration (always recorded) by the individual player’s mean historical PlayerLoad™·min^-1^ for the specific on-court activity completed (e.g., practice, individual on-court work, shootaround, pre-game warmups, game data). Active duration was measured as the time a player was actively engaged in an on-court drill. Breaks in activity, including time transitioning between drills, clock stoppage situations in games and simulated ‘live’ play, and prolonged breaks (i.e., >30 seconds) during drills, were excluded from a player’s active time. The need for thorough descriptions and justifications when reporting duration methods in basketball has been emphasized previously [11], and it is proposed that analyzing physical demands with active duration would allow for practitioners to more accurately calculate intensity demands for basketball activity [11] and inform the development of more precise competition specific training [16]. The mean historical PlayerLoad™·min^-1^ was calculated using all previously measured load data for that individual player, for each specific on-court activity, during the season (i.e., from the beginning of training camp and through the duration of the season). The majority of cases where training load data were estimated included: pre-game individual warm-ups, low intensity ‘shootaround’ activities on game days, and game data for players with an NBA contract [as these players are not permitted to use wearables in games [1]). PlayerLoad™ was summed over the 3 and 10 days preceding the collection of AROMs or CMJ data. The 3-day time frame was selected based on previous work suggesting that neuromuscular status in basketball requires ~72 hours to return to baseline following gameplay [17]. Similarly, the 3- and 10-day periods provided consistent short- and medium-term windows of practice and game data specific to the NBA G-League schedule (3-day: 1.0 ± 0.6 games; 10-day: 3.3 ± 1.1 games).

Recovery days were defined as a day without travel, games, or team practice and minimal (i.e., <200 PlayerLoad™ units) or no on-court activity accumulated by the player. Any minimal on-court work was less than 200 PlayerLoad™ units, which typically represents <45 minutes of light basketball activity [18]. Recovery days were recorded throughout the season and players were defined as either having a recovery day, or not having a recovery day, in the 3 days preceding the collection of AROMs or CMJ data.

Travel data (e.g., approximate hours travelled, and days spent away from the team’s home city) were recorded from flight logs over the course of the season and stored in a customized Microsoft Excel spreadsheet (Microsoft, Redmond, WA, USA) to quantify travel demands. Similar to previous quantifications of in-competition travel [19], hours travelled was defined as the hours between leaving the team’s departure point and arriving at the destination and was summed each travel day and over entire away trips. Overall, 3- and 10-day windows were used to represent cumulative travel stress. Days spent away from the team’s home city was calculated by dividing the total hours away by 24 hours and was summed in the 10 days preceding the collection of AROMs or CMJ data. A 3-day travel time frame was selected based on work suggesting that, when travelling across 2 or more time zones, symptoms of travel fatigue can persist for up to 2–3 days after arrival [5], while the 10-day time frame captured periods of the team schedule that could include one full trip away from the team’s home city and potentially multiple days involving travel.

#### Athlete response measures

AROMs were assessed via a digital survey adapted from the work of McLean et al. [9] and players were asked to complete the survey before team court activities (i.e. 09:00–11:00) on home practice days. AROMs were also collected alongside CMJ data on predetermined game days. Questions related to fatigue, general muscle soreness, and sleep were asked on a ten-point scale with five written anchors. Scores were converted to individual z-scores (i.e., the score in terms of number of standard deviation (SD) units the raw score is above or below the individual’s mean [9]) and these calculations used all data points collected from each player during the season to account for individual fluctuations in perceived responses.

CMJ testing began on the first day of formal training, with 18 assessments performed across 2 seasons (4 pre-season tests; 14 in-season tests) as part of the regular monitoring process of the team. In-season assessments were executed at the home practice facility and before team court activities on predetermined game days (n = 5) and practice days (n = 9). All CMJ data included in this study were collected using protocols we have previously described [8].

CMJ’s were assessed on dual force platforms (ForceDecks FD4000, Vald Performance, Brisbane Australia) sampling at a rate of 1000 Hz. ForceDecks software (Vald Performance, Brisbane Australia; Version 2.0.7188) calculated 105 bilateral force-time CMJ variables for all jumps, via methods previously described [20]. Based off our previous work examining reliability and sensitivity with this cohort [8], and to establish parsimony, we selected 8 CMJ variables that were highly sensitive to changes in athlete status across the NBA G-League season (i.e., seasonal variability was greater than within-subject variability): countermovement depth, eccentric braking rate of force development (RFD), eccentric duration, eccentric mean deceleration force, mean eccentric and concentric power over time, eccentric deceleration phase duration, eccentric peak power and eccentric peak velocity. Final data were checked for outliers and all data points were within 3 SD above or below the individual’s mean.

#### Development of conceptual framework

**Fig 1:**
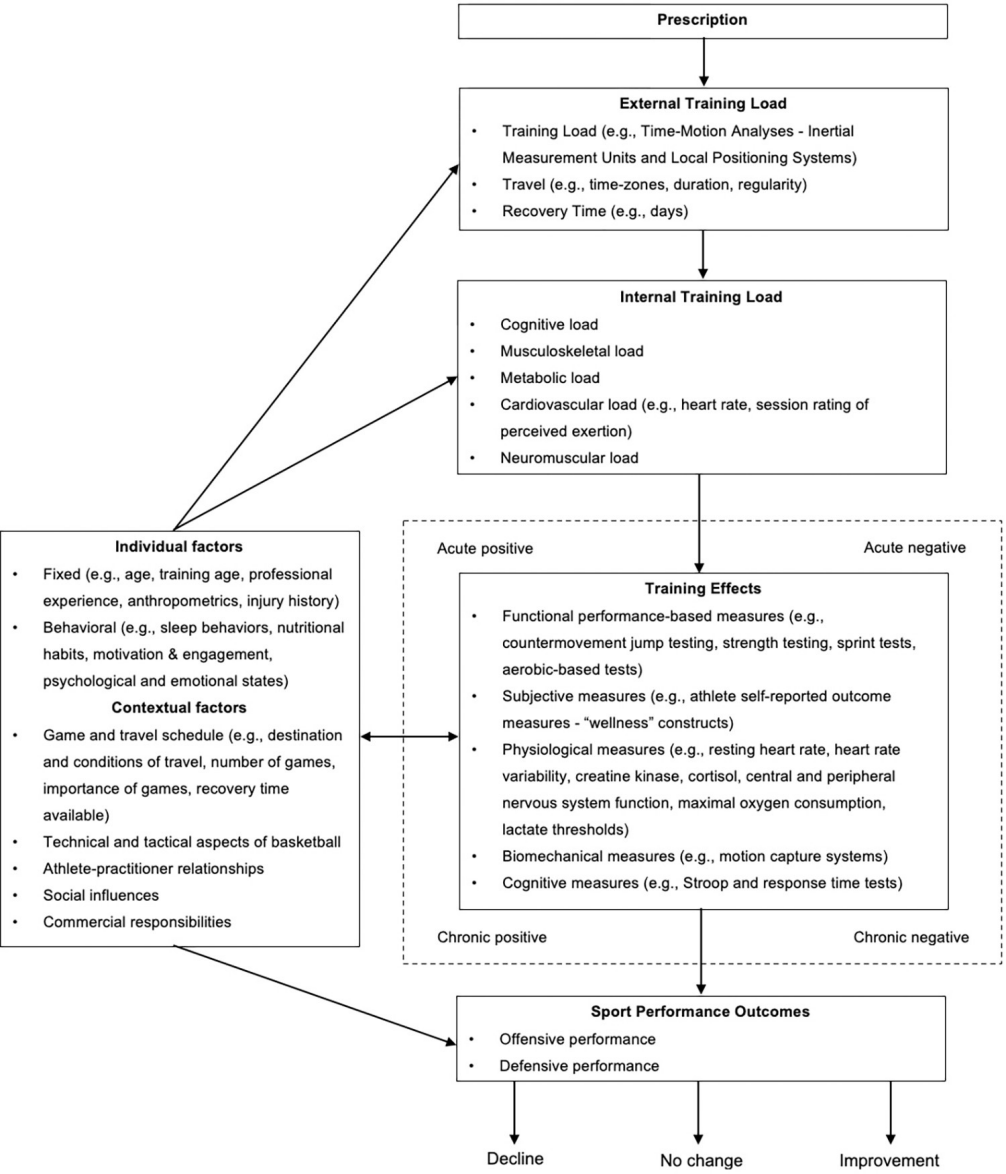
Conceptual framework for physical training in professional basketball, adapted from the work by Jeffries et al. [12] Prescription represents the short and long-term planning and execution of training, competition and travel over the course of the season (i.e., nature and organization of training sessions and travel). External load represents the physical demands associated with training, competition, and travel during the season, and training load is the specific stimulus induced by both training sessions and competition. Internal load represents the psychophysiological responses occurring during the execution of training. Contextual factors are defined as factors that are not part of the main training process, such as environmental, social, and cultural factors, but can influence the training process or outcome. Individual factors are characteristics of the individual athlete, such as genetics, psychological traits and states, and training background, which can influence the training process or outcome. Training effects can be acute or chronic, and positive or negative, effects caused and occurring after the training session, and can be assessed using functional, subjective, physiological, biomechanical and cognitive measures. The bidirectional arrow represents a reciprocal nature of interactions between training effects and individual/contextual factors. For example, a negative training effect (e.g., increased fatigue or poor sleep) can act as an individual factor influencing the internal training load in the subsequent session. Sports performance outcomes are defined as the result of the balance between positive and negative training effects.

The conceptual framework (Fig 1) was theorized using information and constructs (e.g., external and internal load, individual and contextual factors, training effects and sports performance outcomes) adapted from previous models for physical training [12], stress-related, strain-related and overuse athletic injuries [21], and temporal relationships between exercise, recovery processes and changes in performance [4]. The constructs within the framework were then informed using previous literature in basketball regarding the measurement of physical demands [1, 11], external and internal training load models [22, 23], monitoring fatigue and neuromuscular performance [24, 25], travel [5, 26] and recovery [6]. This information was supplemented with practical experience and expert consultation within the research team. Measurable components in the conceptual framework were identified in combination with the available assessment tools and technologies used within the environment (e.g., training load data, travel data, AROMs and CMJ measures). Similarly, suitable time frames of training load and travel were considered, based on previous literature and the research team’s experience in practice, to provide consistent short- and medium-term windows of practice, game, and travel data for the G-League environment.

### Statistical analyses

Twelve separate 2-level linear mixed models were used to examine associations between training load, travel, recovery days, and individual factors with AROMs and CMJ performance over the course of the season (Tables 1 and 2). The study design located units of analysis (individual season samples) nested in clusters of units (players). This form of analysis may contain both fixed effects (effects that describe the association between a dependent variable and covariates for a population) and random effects (effects associated with a random factor, usually signifying random deviations from relationships described by fixed effects) [27]. In the event there were no random effects for individual players (i.e., no significant variance at level 2), a multiple linear regression using a stepwise approach was used to determine the influence of fixed effects only on the dependent variable (i.e., AROMs or CMJ measures). Simple descriptive statistics (mean ± SD) and Pearson correlations were calculated with the multiple linear regression coefficients.

**Table 1:** Covariates included in model specification for athlete-reported outcome measures.

Data Level		Factors	Type	Classification
Level 2	Cluster of units (random factor)	Player		
	Covariate	Age	Dummy variable	0 = 20–25 (n = 12), 1 = 26+ (n = 11)
Level 1	Unit of analysis	Individual season samples		
	Dependent variable	Fatigue	Continuous	AU–z-score
		Soreness	Continuous	AU–z-score
		Sleep quality	Continuous	AU–z-score
		Sleep hours	Continuous	AU–z-score
	Covariates	Accumulated training load (3-day)	Continuous	AU
		Accumulated training load (10-day)	Continuous	AU
		Hours travelled (3-day)	Continuous	hours
		Hours travelled (10-day)	Continuous	hours
		Days away from home city (10-day)	Continuous	days
		Recovery days (3-day)	Dummy variable	0 = no, 1 = yes

Abbreviations: AU, arbitrary units.

**Table 2:** Covariates included in model specification for countermovement jump measures.

Data Level		Factors	Type	Classification
Level 2	Cluster of units (random factor)	Player		
	Covariate	Age	Dummy variable	0 = 20–25 (n = 12), 1 = 26+ (n = 11)
		Fatigue	Continuous	AU—z-score
		Soreness	Continuous	AU—z-score
Level 1	Unit of analysis	Individual season samples		
	Dependent variable	Countermovement Depth	Continuous	cm
		Eccentric Braking RFD	Continuous	N/s
		Eccentric Duration	Continuous	ms
		Eccentric Mean Deceleration Force	Continuous	N
		Mean Eccentric+Concentric Power:Time	Continuous	W/s
		Eccentric Deceleration Phase Duration	Continuous	s
		Eccentric Peak Power	Continuous	W
		Eccentric Peak Velocity	Continuous	m/s
	Covariates	Accumulated training load (3-day)	Continuous	AU
		Accumulated training load (10-day)	Continuous	AU
		Hours travelled (3-day)	Continuous	hours
		Hours travelled (10-day)	Continuous	hours
		Days away from home city (10-day)	Continuous	days
		Recovery days (3-day)	Dummy variable	0 = no, 1 = yes

Abbreviations: AU, arbitrary units; CMJ, countermovement jump; RFD, rate of force development.

Random factors were included in the model to investigate deviations for players from the overall fixed intercept and fixed coefficients. The *t* statistic and degrees of freedom (*df*) calculated for the linear mixed model were converted to get an effect size correlation (*r*) between each factor and scores on the dependent variable [28]. These effect size correlations were interpreted as < .1, trivial; .10-.29, small; .30-.49, moderate; .50-.69, large; .70-.89, very large; .90-.99, almost perfect; 1.0, perfect [29]. A “step up” model construction strategy was implemented, beginning with an “unconditional” model containing only a fixed intercept and Level 2 random factors to determine if variation existed in the dependent variable and to establish an initial Akaike’s Information Criteria (AIC) [27]. The models were then developed by adding single level 1 fixed effects, followed by level 2 fixed effects. The order in which the variables were added was determined by factors that were likely important, based on previous research and the investigation team’s own experience in the field. Single fixed effects were retained in the model if it displayed statistical significance (*p* < 0.05) and contributed to model fit by improving the information criteria (i.e., AIC) compared to the previous model. The intraclass correlation coefficient (ICC) was used to determine the similarity of observed responses within the individual player clusters. Final model’s residuals were visually inspected for normality. All statistical analyses were conducted using IBM SPSS Statistics Subscription (build 1.0.01508).

## Results

### External load measures

#### Training load and recovery

Over 2 seasons, 2,784 hours of on-court data were collected (i.e., training load data). This included 2,435 hours (87%) where training load was quantified using IMU’s and 348 hours (13%) where players did not wear an IMU, and load was estimated from measured duration and historical activity data. Most estimated load data were from shootarounds (4% total data), pre-game warmups (4% total data) and game data for two-way players (n = 3 players, 2% total data). The players had a mean cumulative 3-day training load of 834 ± 449 arbitrary units (AU) (range: 500–1319) and a mean cumulative 10-day training load of 2722 ± 1018 AU (range: 1640–4378). Players had a mean of 29 ± 4 recovery days during the season, with a mean of 6 ± 4 days between them (range: 0–17 days). Frequencies of recovery days across all players for AROM and CMJ collections can be seen in Table 3.

**Table 3:** Frequencies of recovery days during previous 3 days across all players.

	AROMs (n = 913)		CMJ (n = 182)	
	n	%	n	%
No recovery (0)	399	44%	81	45%
Recovery (1)	514	56%	101	55%

Abbreviations: AROM, athlete-reported outcome measures; CMJ, countermovement jump.

*Travel*. Travel included 29 trips away from the team’s home city across both seasons, with a mean of 3 ± 2 days per trip (range: 1–8 days). There was a mean of 7 ± 4 days between away trips (i.e., days between returning to home city and departing for next trip) and 4 ± 3 days between travel throughout the season. Travel involved a mean of 4.0 ± 1.9 hours travelled per travel day (range: 1.3–8.2 hours travelled) and 10.1 ± 4.6 hours travelled total per away trip (range: 3.9–19.6 hours travelled).

### Athlete response measures

#### AROMs

The digital survey was completed by all players who were present and participated in team activities (i.e., involved during team practice or shootaround) on home practice and select home gamedays across both seasons, resulting in 913 data points across all players (mean ± SD = 36.5 ± 8.0 surveys per player). There were no random effects for individual players for any AROMs. Therefore, a multiple linear regression using a stepwise approach was used to determine the influence of fixed effects only on fatigue, soreness, sleep quality and sleep hours. Simple descriptive statistics and correlations are displayed in Table 4. Final model statistics and regression coefficients for estimating AROMs are displayed in Table 5.

**Table 4:** Means, SD’s and Pearson correlations among athlete-reported outcome measures and independent variables.

Variable	Mean ± SD	1	2	3	4	5	6	7	8	9	10
1. fatigue—z-score	-0.015 ± 0.975	-	.643**	.382**	.234**	-.337**	-.116**	-0.014	.078*	.098**	.124**
2. soreness—z-score	0.001 ± 0.975		-	.347**	.213**	-.303**	-.085**	-0.005	0.057	.071*	.159**
3. sleep quality—z-score	0.000 ± 1.006			-	.527**	-.184**	-.083*	0.042	.086**	.068*	0.062
4. sleep hours—z-score	-0.004 ± 0.998				-	-.146**	-.072*	0.037	0.055	.082*	-0.019
5. Accumulated training load 3-day	801 ± 432					-	.535**	0.050	-0.046	-.083*	-.218**
6. Accumulated training load 10-day	2,606 ± 1,069						-	.216**	.271**	.244**	.081*
7. Hours travelled 3-day	1.50 ± 2.66							-	.578**	.450**	-.257**
8. Hours travelled 10-day	6.14 ± 6.14								-	.827**	.100**
9. Days away from home city 10-day	2.3 ± 2.2									-	.102**
10. Recovery days 3-day	0.56 ± 0.50										-

*p < 0.05

**p < 0.01. Abbreviations: SD, standard deviation.

**Table 5:** Regression coefficients for estimating athlete-reported outcome measures during the season.

	B	95% CI	β	t	*p*
Fatigue—z-score					
(Constant)	0.468	0.306, 0.630	-	5.679	<0.001
Accumulated training load 3-day (AU)	-0.001	-0.001, -0.001	-0.385	-10.445	<0.001
Accumulated training load 10-day (AU)	0.00008	0.000, 0.000	0.090	2.444	0.015
F(2, 910) = 61.469, p < 0.001, R^2^ = 0.119 (n = 913)					
**Soreness—z-score**	** **		** **	** **	** **
(Constant)	0.310	0.132, 0.489	-	3.417	<0.001
Accumulated training load 3-day (AU)	-0.001	-0.001, -0.001	-0.332	-8.503	<0.001
Recovery days 3-day	0.156	0.028, 0.283	0.079	2.393	0.017
Accumulated training load 10-day (AU)	0.00008	0.000, 0.000	0.086	2.248	0.025
F(3, 909) = 35.92, p < 0.001, R^2^ = 0.106 (n = 913)					
**Sleep quality—z-score**	** **		** **	** **	** **
(Constant)	0.259	0.107, 0.411	-	3.342	0.001
Accumulated training load 3-day (AU)	0.000	-0.001, 0.000	-0.181	-5.554	<0.001
Hours travelled 10-day (hrs)	0.013	0.002, 0.023	0.078	2.396	0.017
F(2, 910) = 18.949, p < 0.001, R^2^ = 0.040 (n = 913)					
**Sleep hours—z-score**	** **		** **	** **	** **
(Constant)	0.182	0.026, 0.338	-	2.286	0.022
Accumulated training load 3-day (AU)	0.000	0.000, 0.000	-0.141	-4.283	<0.001
Days away from home city 10-day (days)	0.032	0.003, 0.061	0.070	2.136	0.033
F(2, 910) = 12.303, p < 0.001, R^2^ = 0.026 (n = 913)					

Note: F statistic, degrees of freedom (*df*), R^2^ and *p* values are from the final model. Abbreviations: AU, arbitrary unit; CI, confidence interval for B.

#### Countermovement jump measures

CMJ testing was completed by all healthy (i.e., without illness and injury free, as determined by team staff) and present players on 18 days across both seasons, resulting in 183 assessments across all players (mean ± SD = 7.3 ± 2.2 assessments per player). Initial null modeling showed significant variability (p < 0.001) in all 8 CMJ variables analyzed. For all the models, the construction process was optimized by including a random intercept effect for individual players, showing that there was statistically significant variance between individual players for all 8 CMJ measures. The ICCs for individual season samples within each player were 0.60, 0.86, 0.58, 0.89, 0.12, 0.15, 0.85 and 0.17 for Countermovement Depth, Eccentric Braking RFD, Eccentric Duration, Eccentric Mean Deceleration Force, Mean Eccentric+Concentric Power:Time, Eccentric Deceleration Phase Duration, Eccentric Peak Power, and Eccentric Peak Velocity, respectively. There were no random coefficient effects for any level 1 covariates in any of the final models, indicating that the effects shown were consistent across individual players for each countermovement jump measure. Simple effects for all countermovement jump measures are shown in Table 6.

**Table 6:** Simple effects for countermovement jump measures.

	Coefficient estimate	Standard Error	95% CI	df	t	*p*	Effect size (r)
**Countermovement Depth**							
Intercept (cm)	-31.7	-1.2	-34.1, -29.4	21.9	-27.5	<0.001	
Soreness (AU—z-score)	-0.641	0.325	-1.282, 0.001	152.8	-2.0	0.050	0.16
Hours travelled 3-day (hrs)	-0.548	0.198	-0.939, -0.158	150.1	-2.8	0.006	0.22
**Eccentric Braking RFD**							
Intercept (N/s)	4535	461	3,589, 5,480	26.6	9.8	<0.001	
Accumulated training load 10-day (AU)	0.208	0.062	0.085, 0.330	136.7	3.4	0.001	0.28
**Eccentric Duration**							
Intercept (ms)	621	25	570, 671	32.1	25.2	<0.001	
Accumulated training load 3-day (AU)	-0.037	0.015	-0.068, -0.007	154.2	-2.5	0.015	0.19
**Eccentric Mean Deceleration Force**							
Intercept (N)	1505	54	1,394, 1,615	25.7	27.9	<0.001	
Accumulated training load 10-day (AU)	0.021	0.007	0.008, 0.034	139.8	3.2	0.002	0.26
**Mean Eccentric+Concentric Power:Time**							
Intercept (W/s)	1565	106	1,347, 1,783	26.4	14.8	<0.001	
Accumulated training load 10-day (AU)	0.042	0.017	0.008, 0.077	50.6	2.5	0.017	0.33
**Eccentric Deceleration Phase Duration**							
Intercept (s)	0.213	0.011	0.189, 0.236	26.2	18.6	<0.001	
Accumulated training load 10-day (AU)	-0.000005	0.000002	-0.000008, -0.000002	74.6	-3.2	0.002	0.35
**Eccentric Peak Power**							
Intercept (W)	1413	112	1,180, 1,645	23.3	12.6	<0.001	
Days away from home city 10-day (days)	23.276	8.589	6.287, 40.264	132.6	2.7	0.008	0.23
**Eccentric Peak Velocity**	** **	** **	** **	** **	** **	*** ***	** **
Intercept (m/s)	-1.116	0.049	-1.218, -1.014	22.2	-22.6	<0.001	
Hours travelled 3-day (hrs)	-0.013	0.006	-0.025, -0.001	149.2	-2.2	0.033	0.17

Abbreviations: AU, arbitrary unit; CI, confidence interval for coefficient estimate.

## Discussion

The primary finding of the present study was that AROMs and CMJ measures are associated with fluctuations in training load and travel demands over the course of a professional basketball season. These measures are elements of the constructs of external load and training effects, encapsulated in the conceptual framework that we developed (Fig 1) to better understand physical training in professional basketball. Specifically, cumulative training load over 3 and 10 days, recent travel time and days spent away from the team’s home city were all associated with changes in CMJ performance during the eccentric phase. Similarly, cumulative 3-day training load had a significant negative association with all subsequent AROMs (i.e., fatigue, soreness, sleep quality and sleep hours), while a recovery day in the previous 3 days had a significant positive association with soreness scores. An increase in hours travelled and days spent away from the team’s home city in the previous 10 days was also associated with significantly improved sleep quality scores and self-reported sleep hours. The current work has taken a novel approach in developing and applying a conceptual framework to inform context specific training monitoring in basketball [12]. Using this approach, we were then able to highlight context specific associations between physical demands in the NBA G-League and common athlete response measures used to estimate training effects across the season.

### Training load demands and recovery days

The current findings show negative associations between cumulative training load and several AROMs, which highlights the close relationship between prescribed training load and athlete responses. Our results suggest that it is important to consider both short- (i.e., 3-day) and medium-term (i.e., 10-day) training load when developing individualized training plans, as both these load constructs were associated with increases in perceptual responses of fatigue and soreness. While there are similar relationships between these two load epochs and AROMs, cumulative 3-day training load produced stronger effects (i.e., larger absolute standardized β coefficient–see Table 5) for perceptual fatigue and soreness and had an impact on a greater number of AROMs (i.e., increases in short-term training load were also associated with decreases in sleep quality and sleep hours). This suggests that AROMs are more responsive to short-term changes in training load, compared to cumulative 10-day training load. However, medium-term training load should not be discounted in the monitoring and individualization of the planning process, given its association with fatigue and soreness responses in the present work. Moreover, it has previously been shown that periods of intensified training and competition can lead to an accumulation of exercise-induced muscle damage, accompanied by decreases in neuromuscular function, increased perceptual fatigue and soreness, and reductions in sleep duration and sleep quality [30, 31]. As increased training loads can disrupt the stress-recovery balance (i.e., the balance between training stress and subsequent rest), it is likely important to understand both short- and medium-term training load, to optimize training prescription and avoid unwanted performance decrements and potential increases in injury incidence [32].

Neuromuscular function (e.g., fatigue, supercompensation, de-training) is commonly used to assess athletes during the season, including acute responses and chronic adaptations to training and competition [33]. We have previously reported the reliability and sensitivity (i.e., seasonal variability greater than the within-subject variability) of a CMJ assessment in professional basketball and showed a large number of variables have greater seasonal variability than the inherent noise [8]. However, there is a paucity of data investigating potential relationships with CMJ performance and physical demands experienced across a professional basketball season [8]. The present results show that accumulated training load is associated with changes in several CMJ measures. Indeed, we found that increases in cumulative 10-day training load were associated with small increases to Eccentric Braking RFD, and increases in cumulative 3-day training load were associated with small decreases in Eccentric Duration, which is thought to be an important indicator of stretch reflex sensitivity and overall eccentric function [33]. In contrast to our AROM results, it appears that CMJ measures are more responsive to changes in medium-term (i.e., 10-day), versus short-term (i.e., 3-day), training load. Indeed, 4 of 8 CMJ measures investigated were associated with cumulative 10-day training load, with effect size correlations ranging from small to moderate (range: *r* = 0.26–0.35), compared to only 1 variable associated with cumulative 3-day training load (*r* = 0.19). Collectively, these findings highlight the association between medium-term cumulative training load and CMJ measures used to estimate neuromuscular status (e.g., fatigue, supercompensation, de-training) across the season, and supports the collection of these measures, as components of a sensitive and responsive training system, to optimize individual training prescription.

While there has been a significant amount of work performed with outdoor tracking technologies (e.g., global positioning systems) in other team sports, these findings cannot be applied to indoor sports [1]. Indoor technologies (e.g., local positioning systems) have become more ubiquitous recently, creating the potential for more complete training load data sets (i.e., game and practice data) to be collected in basketball moving forward. In the present work, we directly measured 97% and 98% of all practice and game data (excluding game data for NBA contracted players), respectively, across 2 seasons. Such consistent collection of training load data is important to elucidate ecologically valid associations between physical demands and athlete response measures, and subsequently support the development of individualized and context-specific training planning solutions. It is also important to consider suitable training periods that provide information relevant to the desired performance outcome and training effect measures [34], as characteristics unique to those time frames likely impact athlete response. These nuances in training response highlight the importance of underlying evidence which supports decision-making. Indeed, the training periods considered in this study (i.e., 3- and 10-day cumulative training load) were chosen by evaluating basic concepts of training adaptation with individual, contextual, and environmental factors specific to performance within the NBA G-League.

Given that increased training loads during the season can disrupt the balance between training stress and subsequent rest [32], it is important to consider the prescription of adequate recovery time (e.g., recovery days with minimal activity and no travel) when developing a training plan. We showed that having a recovery day in the previous 3 days was associated with improved perceptual soreness responses over the course of the season. Conversely, recovery days were not significantly associated with fatigue scores, which suggests that while recovery days can alleviate perceptual feelings of soreness, a single recovery day at given intervals (i.e., every 5.5 ± 3.9 days during the season) is not solely enough to mitigate cumulative fatigue during the season. Thorpe et al. [35] showed that, when monitoring fatigue throughout an in-season training week in soccer, there was no significant improvement in perceived ratings of fatigue from day 2 to day 4 after a match, despite a recovery day scheduled on day 3. They suggested that the magnitude of training load assigned on day 2 provided sufficient stimulus to blunt a linear improvement in player fatigue on day 4 [35]. While caution should be taken when comparing the time course of recovery between soccer and basketball (e.g. differences in physical demands and competition/practice schedules [17]), our results support the suggestion that consistent on-court loading throughout the season prevents players from gaining significant improvements in perceived ratings of fatigue following recovery days. Similarly, despite 55% (n = 101) of the CMJ assessments being performed after having a recovery day in the previous 3 days, the present results show no evidence of significant associations between recovery days and CMJ measures. The absence of significant improvements to both subjective (e.g., fatigue and sleep) and functional performance-based measures (e.g., CMJ measures) following recovery days suggests that frequent on-court loading and extensive travel prevent players from fully recovering from the accumulated physical and psychological stress throughout the season and this may influence sports performance outcomes [5].

### Travel

The condensed travel schedule required during an NBA G-League season likely predisposes the players to travel-induced fatigue, which can include feelings of disorientation, lack of energy, and general discomfort, and could impact performance during the season [5]. In the present study, the hours travelled, and days spent away from the team’s home city in the previous 10 days were associated with increased sleep quality and sleep hours, respectively, which suggests that players require additional sleep to recover from the “travel fatigue” accumulated. These results are in contrast to previous research, which reported that frequent travel can negatively affect both sleep quality and sleep hours [36]. The present findings may be specific to the rhythm of a G-League schedule, as periods of intensified travel within the last 10 days are usually followed by a period of home games. The combination of disruptive travel periods immediately followed by a return to players’ own homes and beds may create this unique circumstance of recent travel demands being associated with improved sleep indices. However, there were no instances of AROMs collected when away from the team’s home city and as such, it is difficult to directly compare the players’ sleep quality and sleep hours when travelling with the team. Nevertheless, it is important for practitioners to consider travel demands when developing sensitive and responsive training systems for their environment. For example, practitioners could use this information to adjust training schedules (e.g., later treatment and practice times) or implement strategies to promote sleep when players are in their home city, particularly after periods of increased travel.

Travel was also associated with CMJ measures over the course of the season, with an increase in the hours travelled in the previous 3 days being related to small increases to both Countermovement Depth (*r* = 0.22) and Eccentric Peak Velocity (*r* = 0.17). Furthermore, an increase in days spent away from the team’s home city in the previous 10 days was associated with small increases (*r* = 0.23) to Eccentric Peak Power. It has been suggested that variability in eccentric variables is primarily driven by changes in technique related to the speed and depth of the countermovement [37], and our previous work highlighted that Countermovement Depth and Eccentric Peak Velocity were two of the most responsive variables across the season in this cohort [8]. Skilled jumpers are also thought to be capable of adjusting strategy to maintain output [20], therefore it is possible that these CMJ changes highlight instances of athletes using an alternate strategy to maintain CMJ output [33], after physically demanding travel periods. In support of this idea, previous research has suggested that an increase in Eccentric Peak Velocity, similar to that seen following travel in the present work, serves to limit the concentric-performance decrement seen 72 hours following a fatiguing protocol meant to simulate team-sport activities [33].

In addition to the impact from the physical act of travelling, the effects of circadian misalignment and time zone changes are also an important consideration [5, 38]. Indeed, negative associations between the number of time zones crossed and mood have previously been reported in elite athletes [39], while studies in the NBA found that westward travel negatively impacted winning percentages [26, 40]. However, previous research has commonly separated travel-induced fatigue from jet-lag fatigue, with the main difference being that jet-lag fatigue comprises an effect of time zone change while travel fatigue is driven by factors such as regularity, duration and conditions of travel [41]. The current results suggest that reporting measures such as travel duration (e.g., hours travelled and days spent away from the team’s home city) with the number of time zones crossed may be suitable for estimating the training effects (e.g., both travel-induced and jet-lag fatigue) associated with travel over the course of the season. However, other factors such as the timing of travel (i.e., time of day travel is completed) are important to consider when planning travel schedules as travel timing and conditions can vary greatly across the season and between competitions. In the NBA G-League, teams travel primarily via commercial flights, but long duration (i.e., > 6 hours) bus travel through the night (i.e., after midnight) is also common, and this could result in a ‘time-zone’ style disruption for the players despite minimal changes in time-zone. Ultimately, improving understanding around all physically demanding aspects of the travel schedule (i.e., regularity, duration, conditions, timing, time-zone changes) is important to optimize travel and training planning solutions, and to mitigate any negative effects throughout the season.

### Individual factors

To investigate the influence of individual factors on training effects, we considered the player’s age and perceived fatigue/soreness as individual factors that may influence other AROMs and CMJ measures during the season. Jeffries et al. [12] highlighted that negative training effects (e.g. increased fatigue) can act as an individual factor subsequently influencing the training response (e.g. causing higher or lower negative effects). However, there were no significant associations between the individual factors included in these models and any of the AROMs and CMJ measures investigated. The only association that approached significance (p = 0.050) suggested a decrease in soreness scores (i.e., increased perceptual feelings of soreness) was associated with a small reduction in Countermovement Depth (*r* = 0.16). Limited countermovement during the jump test may be another example of players adjusting their movement strategy to maintain performance output, when they are experiencing increased soreness. It is also important to acknowledge that player’s individual behaviors (e.g., sleep hygiene and nutritional habits) not measured in this study could also affect how they respond to training, travel, and recovery days during the season. However, further research is required to elucidate associations between individual factors (e.g., fixed, and behavioral) and training effect measures.

### Limitations

Identifying factors that are significantly associated with training effects assessed during the season can improve our understanding and help to inform and optimize individual preparation and periodization strategies, however there is still a lot of variability left unexplained. Similarly, previous literature has suggested that AROMs presented as single use items are more prone to misinterpretation [42]. However, the constructs assessed in the AROM survey were unidimensional in nature and included unambiguous verbal anchors which can yield acceptable validity [42]. Another limitation is that training load data were estimated for limited cases (i.e., 13% of all on-court activity over 2 seasons) where players did not wear an IMU for a session. However, we directly measured 100% of all on-court duration (i.e., only the load derived from IMU data was estimated) and most estimated load data were for low intensity ‘shootarounds’ (4% total data) and pre-game warmups (4% total data). An additional 2% of total data could not be collected, as players with NBA contracts (i.e. ‘two-way’ players) are not permitted to wear an IMU during games [1]. Indeed, across two seasons we directly measured 97% and 98% of all practice and game load data, respectively (excluding data for NBA ‘two-way’ players, n = 3 players). While having zero estimated load data is ideal, this is considered impossible in practice [43] and the statistical approach (i.e., linear mixed models) used in this investigation was chosen based on its ability to account for missing data in the analysis. We believe that the consistency of the data used, including two cohorts over two seasons, supports the interpretation of the findings in this study and should be considered a strength of this work. However, the current work does not evaluate all the potential physical demands (e.g., timing and conditions of travel, resistance training) and individual (e.g., injury history, sleep behaviors, nutritional habits) or contextual factors (e.g., social influences, commercial responsibilities) that could be associated with training effects, nor does it examine other potential training effect measures (e.g., physiological, biomechanical, or cognitive measures). Indeed, we suggest that practitioners interpret previous work that claims to predict or explain training effects based on singular measures of physical demands (e.g., training load) with caution, as such claims are not acknowledging many contextual factors.

Measuring all factors that are important in our conceptual framework (and those we may have overlooked in our framework) of basketball performance is impossible, but we have shown that various measures are interrelated and can potentially provide value in the training prescription process. A continued evolution of the scientific evidence in this area will facilitate better understanding around the measures/variables that explain the greatest amount of variance in training responses, within a given context (e.g., NBA G-League season). This information can aid practitioners in developing parsimonious feedback structures which meaningfully inform individualized training prescription and periodization.

The present work enhances our understanding of associations between physical demands and training effects measured in professional basketball, with various aspects of training load and travel affecting athlete response measures throughout the season. The interaction of these stressors combined with individual factors is complex, meaning that multiple athlete response measures are needed to understand fatigue and recovery cycles even partially. This study also highlights the utility of presenting a conceptual framework to help synthesize evidence, assist in understanding phenomena, inform future research and act as a reference operational guide in practical settings [12]. Indeed, the current findings provide ecologically valid information clarifying the utility and validity of the monitoring tools used to assess external load and training effects for this environment, which fit within the conceptual framework we have presented. However, this is not an exhaustive monitoring battery and practitioners should consider what context-specific measures may best inform training periodization for their environment. Ultimately, by enhancing our understanding of the relationships between external load (e.g., training load and travel), recovery, training effects and sports performance outcomes, we support the development of sensitive and responsive training systems [10] and inform best-practice models for athlete care and performance in professional basketball [1].

### Practical applications

Several measures presented in this work are useful in understanding athlete responses related to external load throughout a basketball season. Therefore, practitioners should consider using these variables (i.e., training load, travel duration, AROMs, CMJ measures) or similar measures to inform planning and prescription throughout the season. However, no single measures of external load or athlete response can explain all the variance involved in the training process and it is likely that the most valid and sensitive variables are highly contextual. Therefore, practitioners should develop their own holistic, conceptual models of performance that are specific to a given environment, to inform the selection of monitoring tools which assess different external load and athlete response constructs. While this approach will inform best-practice periodization, even the most valid and complete athlete monitoring systems cannot quantify all factors that are important in sports performance. Therefore, practitioners should use these systems to inform individualized prescription, but also consider factors that are not quantified during the decision-making process.
